# Interpersonal eye-tracking reveals the dynamics of interacting minds

**DOI:** 10.3389/fnhum.2024.1356680

**Published:** 2024-03-12

**Authors:** Sophie Wohltjen, Thalia Wheatley

**Affiliations:** ^1^Department of Psychology, University of Wisconsin–Madison, Madison, WI, United States; ^2^Department of Psychological and Brain Sciences, Consortium for Interacting Minds, Dartmouth College, Hanover, NH, United States; ^3^Santa Fe Institute, Santa Fe, NM, United States

**Keywords:** eye-tracking, social attention, gaze, blinking, pupillometry, social interaction, naturalistic experimental design

## Abstract

The human eye is a rich source of information about where, when, and how we attend. Our gaze paths indicate where and what captures our attention, while changes in pupil size can signal surprise, revealing our expectations. Similarly, the pattern of our blinks suggests levels of alertness and when our attention shifts between external engagement and internal thought. During interactions with others, these cues reveal how we coordinate and share our mental states. To leverage these insights effectively, we need accurate, timely methods to observe these cues as they naturally unfold. Advances in eye-tracking technology now enable real-time observation of these cues, shedding light on mutual cognitive processes that foster shared understanding, collaborative thought, and social connection. This brief review highlights these advances and the new opportunities they present for future research.

## 1 Introduction

Social interactions form the bedrock of human experience, shaping our emotions, beliefs, and behaviors while fostering a sense of belonging and purpose (Cacioppo and Patrick, 2008). But the mechanics of this crucial behavior remain a mystery: How does the brain activity of one individual influence the brain activity of another, enabling the transfer of thoughts, feelings, and beliefs? Moreover, how do interacting minds create new ideas that cannot be traced back to either individual alone? Only recently has science begun to tackle these fundamental questions, utilizing new methods that can trace the complex dynamics of minds that adapt to each other (Risko et al., 2016). Still far from mainstream (Schilbach et al., 2013; Schilbach, 2016), the science of interacting minds is an growing field. This growth is driven by the realization that relying solely on static models and single-participant studies has constrained our understanding of the human mind (Wheatley et al., 2023).

Tackling these core questions of mind-to-mind influence presents significant challenges. The seemingly effortless and common nature of interaction masks its underlying complexity (Garrod and Pickering, 2004). Even the simplest interaction involves multiple communication channels, leading to the continuous reshaping of thought (Shockley et al., 2009). Commensurate with this complexity, many methodologies struggle to capture the ongoing, reciprocal dynamics. The restrictive environments of neuroimaging machines and concerns of motion artifact (Fargier et al., 2018) make scanning interacting minds challenging (Pinti et al., 2020) while behavioral studies can be laborious and challenging to scale. Other physiological signals, such as heart rate, offer valuable insights into the synchrony between people, a phenomenon that may be amplified by mutual attention (for example, individuals listening to the same story may experience synchronized heart rate variations (Pérez et al., 2021). Nonetheless, these indicators often fall short in providing detailed insights into how individuals coordinate their attention with one another from one moment to the next.

One technique has increasingly surmounted these challenges: eye-tracking. While not explicitly neuroscientific in traditional terms, pupil dilations under consistent lighting are tightly correlated with activity in the brain's locus coeruleus (Rajkowski, 1993; Aston-Jones et al., 1994), the neural hub integral to attention. Fluctuations in pupil size track the release of norepinephrine, providing a temporally sensitive measure of when attention is modulated (Joshi et al., 2016). Further, gaze direction and blink rate offer their own insights into what people find interesting and how they shift their attention between internal thought and the external world. This one technique thus produces multiple sources of information about the mind that are temporally sensitive and can be monitored passively without affecting the unfolding of natural responses.

Interpersonal eye-tracking—eye tracking with two or more individuals—captures the moment-by-moment attentional dynamics as people interact. This technique is already bearing fruit. For example, research has demonstrated its use in detecting the emergence of mutual understanding. When people attend in the same way, their pupil dilations synchronize providing a visual cue of minds in sync (Kang and Wheatley, 2017; Nencheva et al., 2021). Similarly, correspondence between people's gaze trajectories, blink rates and eye contact provide additional cues that reveal how minds interact (Richardson et al., 2007; Nakano, 2015; Capozzi and Ristic, 2022; Mayrand et al., 2023). With the emergence of wearable devices, such as eye-tracking glasses, we can more easily monitor these cues as they unfold naturally during social interactions in ways that are portable across diverse settings, demand minimal setup, and are scalable to larger groups. Coupled with new, innovative analytical techniques, these recent advances have made eye-tracking a portable, inexpensive, temporally precise, and efficient tool for addressing fundamental questions about the bidirectional neural influence of interaction and how these processes may differ in populations that find communication challenging (e.g., Autism Spectrum Conditions). In this mini review, we briefly describe the evolution of this technique and its promise for deepening our understanding of human sociality.

## 2 Major advances in eye-tracking

The first eye-trackers were designed as stationary machines, with a participant's head stabilized by a chin rest or bite-bar, restricting movement and field of view (Hartridge and Thomson, 1948; Mackworth and Mackworth, 1958; Płużyczka and Warszawski, 2018). Later, head-mounted eye-tracking cameras were developed (Mackworth and Thomas, 1962) but remained burdensome, restrictive, and required prolonged and frequent calibration, making them unsuitable for the study of social interaction (Hornof and Halverson, 2002). In recent years, eye-tracking technology has witnessed a rapid evolution. In this section, we will highlight some of these recent developments and explore how they have transformed interpersonal eye-tracking into an indispensable resource for understanding social interaction.

Recent technological progress has enabled the eye-tracking of dyads and groups without disrupting their complex exchange of communicative signals. For example, software innovations now automate calibration (e.g., Kassner et al., 2014), synchronize data from multiple devices (e.g., openSync; Razavi et al., 2022) and simplify the analysis of eye-tracking data collected in naturalistic settings (e.g., iMotions, 2022). These breakthroughs streamline device setup, eliminate the need for intrusive recalibrations, and facilitate analysis of gaze and pupil data in real-time. Packages developed within Python (e.g., PyTrack; Ghose et al., 2020), Matlab (e.g., PuPl; Kinley and Levy, 2022; e.g., CHAP; Hershman et al., 2019), and R (e.g., gazeR; Geller et al., 2020) also streamline preprocessing and analysis of eye movement and pupillometry data. Eye-tracking glasses, designed to be worn like regular glasses, afford a wider range of motion, affording natural facial expressions (Valtakari et al., 2021) and gestures, such as the frequent head nods that regulate interaction (McClave, 2000).

New analysis methods for continuous data are better equipped to handle non-linearity and non-stationarity, making them invaluable for quantifying the real-time interplay between the eyes of interacting dyads and groups. For example, Dynamic Time Warping (Berndt and Clifford, 1994) is a non-linear method often used in speech recognition software for aligning time-shifted signals. This method is useful for capturing alignment in social interactions in a way that accounts for noisy, high-resolution data, leader follower dynamics, or other natural features of social interactions where alignment is present but not precisely time-locked. Recent research has employed this method to measure synchrony between two continuous pupillary time series (a measure of shared attention—Kang and Wheatley, 2017; Nencheva et al., 2021; Fink et al., 2023) as people interact (see Section 3 for a detailed description of this phenomenon). Cross-recurrence quantification analysis (Zbilut et al., 1998) quantifies the shared dynamics between two systems, determining lag and identifying leaders and followers during interactions via their gaze behavior (Fusaroli et al., 2014). Advanced methods now allow scientists to analyze the interactions between multiple individuals' eye-tracking data. Multi-Level Vector Auto-Regression (Epskamp et al., 2024) estimates multiple networks of relationships between time series variables, where variables are nodes in the network and edges represent correlations between variables. This method has been used to quantify the relationships between gaze fixation duration and dispersion (Moulder et al., 2022) as well as eye contact, pupil size, and pupillary synchrony (Wohltjen and Wheatley, 2021). Other advanced methods include cross-correlation and reverse correlation (Brinkman et al., 2017), Detrended Fluctuation Analysis (Peng et al., 1994), and deconvolution (Wierda et al., 2012), with the number of analysis techniques continually increasing.

Software is continually improving and open-source, making synchronization of recordings from different eye-trackers more efficient. When analyzing the correspondence between multiple eye-tracking time series, many different methods exist that can account for the non-linearity of eye-tracking data and leverage the multiple measurements that the device captures. These advances have made it relatively simple to collect and analyze eye-tracking data from multiple interacting people across diverse settings.

## 3 What can we learn from interpersonal eye-tracking?

**Figure 1:**
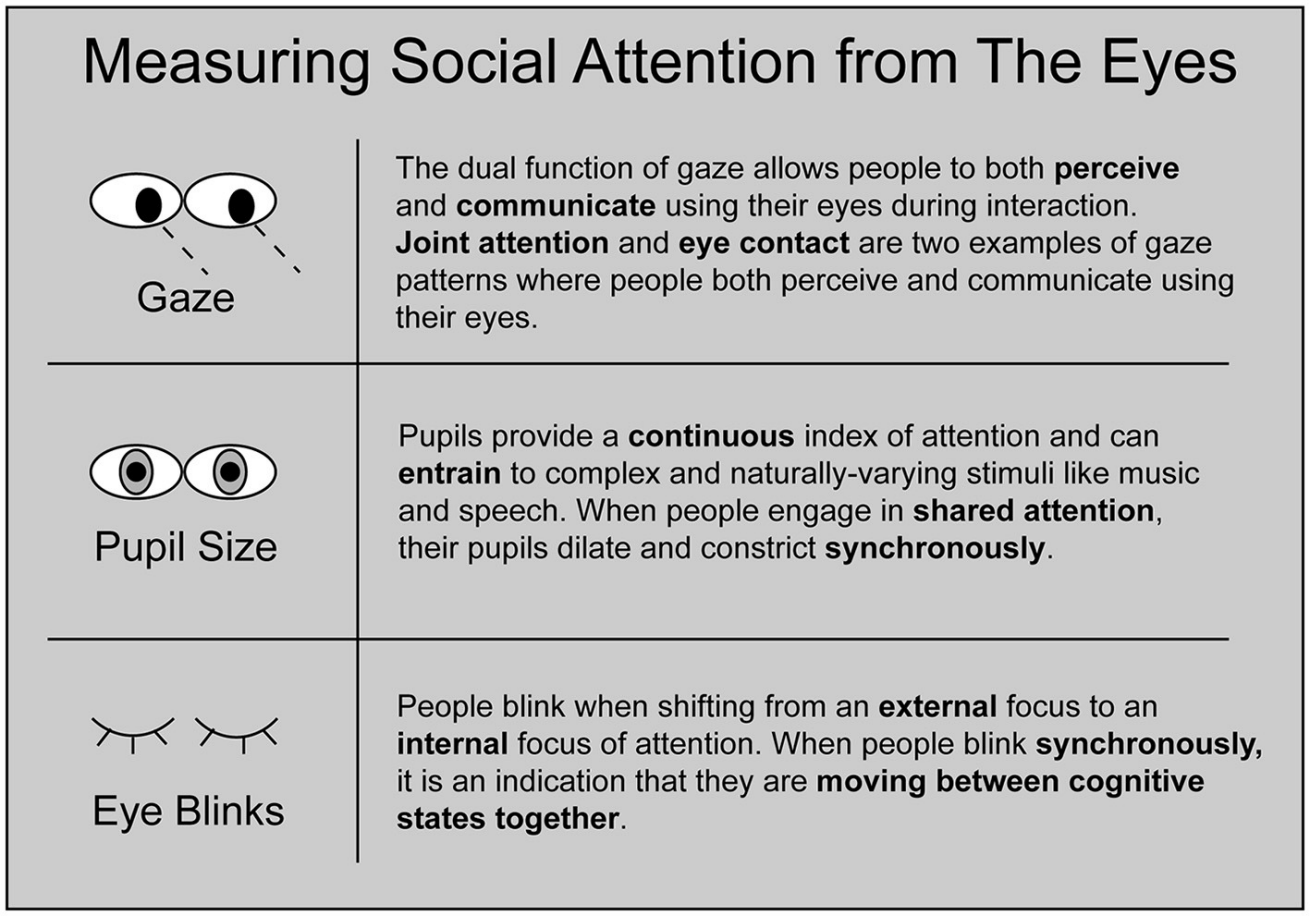
Schematic of three forms of social attention that can be measured using interpersonal eye-tracking. Gaze, pupil size, and eye blinks each reveal unique information about how people dynamically coordinate their attention with each other.

Modern eye-trackers capture several physiological correlates of social attention, including gaze trajectories, pupil dilations, and blink behavior (Figure 1). In this section, we outline how interpersonal eye-tracking leverages these signals to reveal the coordinated dynamics of interacting minds.

### 3.1 Interpersonal gaze: coordinating the “where” of attention

The focus of one's gaze has long been understood as a “spotlight” of attention (Posner et al., 1980), revealing what a person finds most informative about a scene. During social interaction, gaze is often concentrated on the interaction partner's eyes and mouth (Rogers et al., 2018). By virtue of this association, gaze also communicates the focus of one's attention to others (Argyle and Dean, 1965; Gobel et al., 2015).

This dual function of gaze—perception and communication—results in well-established social gaze patterns such as joint attention (Gobel et al., 2015). In joint attention, one person uses their gaze to *communicate* where to look, at which point their partner follows their gaze so that both people are *perceiving* the same thing (Scaife and Bruner, 1975). Joint attention emerges early in life and is critical for the development of neural systems that support social cognition (Mundy, 2017). Another common gaze pattern, mutual gaze (or eye contact), involves a simultaneous process of signaling and perceiving by both parties, embodying a dual role of giving and receiving within the same action (Heron, 1970). Interpersonal eye-tracking provides a unique opportunity to measure both the perceptual and communicative functions of gaze, simultaneously, as they unfold during real interactions. In this vein, a recent study demonstrated that eye contact made people more attentive to the gaze patterns of their conversation partners (Mayrand et al., 2023) and predicted greater across-brain coherence in social brain structures (Hirsch et al., 2017; Dravida et al., 2020). Coupled gaze patterns between conversation partners is associated with their amount of shared knowledge about the conversation topic at hand (Richardson et al., 2007). Thus, attention to another's gaze and the coupling of those gaze patterns appears to indicate engagement and shared understanding.

### 3.2 Interpersonal pupil dilations: tracking when minds are “in sync”

Under consistent lighting, pupil dilations are tightly correlated with the release of norepinephrine in the brain's locus coeruleus (Rajkowski, 1993; Aston-Jones et al., 1994; Joshi et al., 2016), the neural hub integral to attention. When presented with a periodic visual (Naber et al., 2013) or auditory (Fink et al., 2018) stimulus, the pupil dilates and constricts in tandem with the stimulus presentation, which is thought to be the product of prediction (Schwiedrzik and Sudmann, 2020). Pupillary entrainment can also occur with more complex and naturally varying stimuli such as music (Kang et al., 2014; Kang and Banaji, 2020) or speech (Kang and Wheatley, 2017).

When people attend to the same dynamic stimulus in the same way, their pupil dilations synchronize, providing a visual indicator of synchronized minds (Kang and Wheatley, 2015; Nencheva et al., 2021; Fink et al., 2023). For example, when two people listen to the same story, their pupils may constrict and dilate in synchrony indicating that they are similarly anticipating, moment by moment, what will happen next. Synchronized attention, often referred to as “shared attention,” has wide-ranging social benefits including social verification (i.e., the sense that one's subjective reality is validated by virtue of it being shared; Hardin and Higgins, 1996; Echterhoff et al., 2009), heightened perspective-taking (Smith and Mackie, 2016), better memory (Eskenazi et al., 2013; He et al., 2014), and a feeling of social connection (Cheong et al., 2023). This form of synchrony does not require interaction, it is simply a dynamic measurement of how similarly people attend to the same stimulus. It can be measured even when individuals are eye-tracked on separate occasions and are unable to see each other, ruling out pupil mimicry as an underlying cause (Prochazkova et al., 2018).

Recent research investigating pupillary synchrony has uncovered new insights about shared attention. For example, comparing the similarity of toddlers' pupillary dilations as they listened to a story told with child-directed vs. adult-directed speech intonation, Nencheva et al. (2021) found that toddlers had more similar pupillary dilation patterns when hearing child-directed speech, suggesting that it helped entrain their attention. In a conversation study using pupillary synchrony as a metric of shared attention, researchers found that eye contact marked when shared attention rose and fell. Specifically, eye contact occurred as interpersonal pupillary synchrony peaked, at which point synchrony progressively declined until eye contact was broken (Wohltjen and Wheatley, 2021). This suggests that eye contact may communicate high shared attention but also may disrupt that shared focus, possibly to allow for the emergence of independent thinking necessary for conversation to evolve. This may help explain why conversations that are more engaging tend to have more eye contact (Argyle and Dean, 1965; Mazur et al., 1980; Jarick and Bencic, 2019; Dravida et al., 2020).

### 3.3 Interpersonal blink rate: marking changes in cognitive states

When measuring continuous gaze and pupil size, the signal will often be momentarily lost. These moments, caused by blinks, are commonly discarded from eye-tracking analyses yet their timing and frequency are non-random and offer their own clues about the mind (Hershman et al., 2018).

People spontaneously blink every 3 s on average, more than what is necessary for lubricating the eyes (Doane, 1980). Furthermore, the rate of spontaneous eye blinking varies with cognitive states. It changes when chunking information (Stern et al., 1984) or when attending to the rhythmic sequence of presented tones (Huber et al., 2022). Blink rate decreases as attentional demands grow and increases with boredom (Maffei and Angrilli, 2018). However, blink rate also increases with indications of engagement, such as arousal (Stern et al., 1984; Bentivoglio et al., 1997) and attentional switching (Rac-Lubashevsky et al., 2017). Although it seems paradoxical to blink more when bored *and* when engaged, these findings are explained by the role of blinking in the various tasks in which it has been measured. Blinks are related to increased default mode, hippocampal, and cerebellar activity and decreased dorsal and ventral attention network activity. This suggests that blinks may facilitate the transition between outward and inward states of focus (Nakano et al., 2013; Nakano, 2015). As a result, people might blink more frequently when they feel bored due to periodic disengagement, oscillating between focusing on the external environment and their internal thoughts. Additionally, increased blinking occurs in activities requiring regular alternation between external and internal attention, like when participating in conversation (Bentivoglio et al., 1997).

Scientists have discovered intriguing patterns in how people coordinate their eye blinks during interactions. Cummins (2012) observed that individuals strategically adjust their blink rates during conversations based on their partners' gaze direction, indicating shifts between internal and external attention. Moreover, researchers have found that people tend to synchronize their blinks with others during problem-solving tasks (Hoffmann et al., 2023) and conversations (Nakano and Kitazawa, 2010; Gupta et al., 2019), reflecting mutual transitions between cognitive states. Similarly, Nakano and Miyazaki (2019) noted that people who found videos engaging blinked in sync, suggesting shared processing of the content. These studies demonstrate how blinks can signify when interaction partners collectively shift between cognitive states.

Interpersonal eye-tracking records multiple dynamic features that each yield unique insights about how attention is dynamically coordinated and communicated when minds interact (Richardson et al., 2007; Nakano, 2015; Capozzi and Ristic, 2022; Mayrand et al., 2023). Blinking, eye gaze, and pupillary synchrony each reflect dissociable aspects of social attention (see Figure 1). However, these components likely complement and dynamically interact with each other to support social engagement. For example, when pupillary synchrony between conversation partners peaks, eye contact occurs. Coincident with the onset of eye contact, pupillary synchrony declines until eye contact breaks (Wohltjen and Wheatley, 2021). This precise temporal relationship between gaze and pupillary synchrony highlights the importance of combining these measures to shed light on how these components work together as an integrated system.

## 4 Future directions in interpersonal eye-tracking

As interpersonal eye-tracking technology continues to advance, many long-standing questions about interacting minds are newly tractable. In this section, we discuss some of these open research areas, highlighting the untapped potential of interpersonal eye-tracking for the future of social scientific research.

### 4.1 Testing existing theories in ecologically-valid scenarios

Social interaction is immensely complex and difficult to study in controlled laboratory conditions. The ecological validity afforded by interpersonal eye-tracking allows researchers to test how human minds naturally coordinate their attention in ways that afford the sharing and creation of knowledge (Kingstone et al., 2003; Risko et al., 2016). This additional ecological validity is instrumental in discerning the generalizability of psychological theories developed in tightly controlled conditions.

Three recent examples from eye-tracking research highlight potential discrepancies between controlled and naturalistic paradigms. First, a substantial body of research utilizing static images of faces has consistently shown that East Asian participants tend to avoid looking at the eye region more than their Western counterparts (Blais et al., 2008; Akechi et al., 2013; Senju et al., 2013). However, when examined within the context of live social interactions, a striking reversal of this pattern emerges (Haensel et al., 2022). Second, interpersonal eye-tracking has shown that people engage in significantly less mutual gaze than traditional non-naturalistic paradigms would predict. This behavior likely stems from individuals' reluctance to appear as though they are fixating on their interaction partner, a concern that is absent when looking at static images (Laidlaw et al., 2016; Macdonald and Tatler, 2018). Third, interpersonal eye-tracking has revealed how social context can change gaze behavior. For example, when pairs of participants were assigned roles in a collaborative task (e.g., “chef” and “gatherer” for baking), they looked at each more and aligned their gaze faster than pairs who were not assigned roles (Macdonald and Tatler, 2018), suggesting that social roles may help coordinate attention. In the domain of autism research, there has been ongoing debate regarding how autistic individuals use nonverbal cues, such as eye contact. Interpersonal eye-tracking has been proposed to mitigate this lack of consensus by placing experiments in more ecologically valid, interactive scenarios (Laskowitz et al., 2021). With its potential for portability and ease of use, interpersonal eye-tracking offers new opportunities to test existing psychological theories and generate new insights.

Interpersonal eye-tracking also affords direct, side-by-side comparisons of attentional dynamics between tightly controlled lab conditions and more ecologically robust (but less controlled) contexts. For example, Wohltjen et al. (2023) directly compared an individual's attention to rigidly spaced single tones with how well that individual shares attention with a storyteller. Individuals whose pupils tended to synchronize with the tones were also more likely to synchronize their pupil dilations with those of the storyteller. This suggests that the tendency to synchronize one's attention is a reliable individual difference that varies in the human population, manifests across levels of complexity (from highly structured to continuously-varying dynamics) and predicts synchrony between minds.

### 4.2 Tracking moment-to-moment fluctuations in coupled attention

In social interactions, behaviors are dynamic, constantly adjusting to the evolving needs of one's partner and the surrounding context. Techniques that sample a behavior sparsely in time, aggregate over time, or record from a single individual in a noninteractive setting, miss important information relevant to interaction. An illustration of this issue can be found in the synchrony literature, which has long emphasized the benefits of synchrony for successful communication, shared understanding, and many other positive outcomes (Wheatley et al., 2012; Hasson and Frith, 2016; Launay et al., 2016; Mogan et al., 2017). Recent research using interpersonal eye-tracking suggests that synchrony is not always beneficial. Rather, intermittently *breaking* synchrony appears to be equally important (Dahan et al., 2016; Wohltjen and Wheatley, 2021; Ravreby et al., 2022). Mayo and Gordon (2020) suggest that the tendencies to synchronize with one another as well as act independently both exist during social interaction, and that flexibly moving between these two states is the hallmark of a truly adaptive social system. It is possible that several conversational mechanisms prompt this mental state-switching (e.g., topic changes; Egbert, 1997), turn taking (David Mortensen, 2011), and segments of conversation that communicate complete thoughts or Turn Construction Units (Sacks et al., 1978; Clayman, 2012). Future work should investigate how fluctuations of pupillary synchrony, eye blinks and other nonverbal cues help coordinate the coupling-decoupling dynamics between minds that optimize the goals of social interaction.

### 4.3 Tracking the coordinated dynamics of groups

Interpersonal eye-tracking research has traditionally concentrated on dyadic interactions, but the introduction of cost-effective wearable eye-tracking devices has ushered in new possibilities for exploring the intricacies of social interactions within both small and larger groups (Pfeiffer et al., 2013; Cañigueral and Hamilton, 2019; Mayrand et al., 2023). For example, when studying group dynamics, wearable devices allow for the spontaneous head and body movements that naturally occur when interacting with multiple people, such as turning one's head to orient to the current speaker. Recent studies employing wearable or mobile eye-tracking technology in group settings have demonstrated a nuanced interplay of gaze direction, shared attention, and the exchange of nonverbal communication cues (Capozzi et al., 2019; Maran et al., 2021; Capozzi and Ristic, 2022). These studies suggest that interactions are not only about where individuals look but also about the timing and duration of their gaze shifts. Moreover, these studies have highlighted the profound role of gaze as a potent social tool that contributes to the establishment of rapport (Mayrand et al., 2023), the facilitation of group cohesion (Capozzi and Ristic, 2022), and the negotiation of social hierarchies (Capozzi et al., 2019). By increasingly extending the scope of interpersonal eye-tracking research beyond dyads, we stand to gain a more comprehensive understanding of the full spectrum of human social dynamics.

## 5 Discussion

Social interaction is a remarkably intricate process that involves the integration of numerous continuous streams of information. Our comprehension of this crucial behavior has been limited by historical and methodological constraints that have made it challenging to study more than one individual at a time. However, recent advances in eye-tracking technology have revolutionized our ability to measure interactions with high temporal precision, in natural social settings, and in ways that are scalable from dyads to larger groups. The term “eye tracking” belies the wealth of data these devices capture. Parameters such as gaze direction, pupillary dynamics, and blinks each offer unique insights into the human mind. In combination, these metrics shed light on how minds work together to facilitate the sharing and co-creation of thought.

Interpersonal eye-tracking provides exciting opportunities for clinical and educational applications. For example, the relatively low-cost, ease of setup and ability to capture attentional dynamics unobtrusively during interaction make interpersonal eye tracking a promising clinical tool for studying communication difficulties in neurodiverse populations, such as people with Autism Spectrum Conditions (Laskowitz et al., 2021). A recent meta-analysis found that pupil responses in ASC have longer latencies (de Vries et al., 2021), with implications for coordination dynamics in turn-taking. Further, gaze patterns are also diagnostic of ASC from infancy (Zwaigenbaum et al., 2005; Chawarska et al., 2012), with implications for how gaze regulates social interaction (Cañigueral and Hamilton, 2019). Eye-tracking research has also shown that people with aphasia have language comprehension deficits that are partially explained by difficulties in dynamically allocating attention (Heuer and Hallowell, 2015). By using eye-tracking to pinpoint the moments in natural social interaction that increase attention demands, we can learn how social interaction may be adjusted to aid people with attentional difficulties. Interpersonal eye-tracking also has clear implications for understanding how teacher-student and peer-to-peer interactions scaffold learning (e.g., Dikker et al., 2017). We are excited by the accelerating pace of eye-tracking research in naturalistic social interactions that promise to extend our understanding of these and other important domains.

It is important to note that all methods have limitations and eye-tracking is no exception. For instance, changes in pupil size can signal activation in the locus coeruleus, associated with increased attention (Aston-Jones and Cohen, 2005). Yet, this measurement does not clarify which cognitive function benefits from this attentional “gain” (Aston-Jones and Cohen, 2005). Pinpointing a particular mental process or semantic representation, would require incorporating other behavioral assessments, establish comparison conditions, or techniques with higher spatial resolution, such as fMRI. Another challenge arises with the use of wearable eye-tracking technology. While these devices mitigate the issue of signal loss caused by natural head movements, they cannot eliminate it entirely. The freedom of head movement that wearable devices allow can also complicate the interpretation of gaze patterns (such as fixations, quick eye movements, or smooth following movements; Hessels et al., 2020) because gaze is tracked in relation to the head's position.

Despite these challenges, both wearable and stationary eye-tracking technologies offer valuable insights on how people coordinate their attention in real time. The continuous recording of pupil dilations, gaze direction, and blink rate sheds light on the ways that minds mutually adapt, facilitating the exchange of knowledge, shared understanding, and social bonding. By capturing the attentional dynamics of interacting minds, interpersonal eye-tracking offers a unique window into the mechanisms that scaffold social interaction.

## Author contributions

SW: Conceptualization, Writing – original draft, Writing – review & editing. TW: Conceptualization, Funding acquisition, Resources, Writing – original draft, Writing – review & editing.
